# Valorization of Industrial By-Products as a Source of Biopolymers and Active Compounds for the Development of Sustainable Food Packaging and Agronomic Materials

**DOI:** 10.3390/foods15162927

**Published:** 2026-08-20

**Authors:** Luisa Fernanda Sierra Montes, Florencia Ortega, Yuliana Monroy, Florencia Versino, Lorena Deladino, Sandra Rivero, Maria Alejandra García

**Affiliations:** 1 Centro de Investigación y Desarrollo en Ciencia y Tecnología de Alimentos (CIDCA), UNLP-CONICET-CICPBA, 47 y 116, La Plata 1900, Argentina; luisaf.2294@gmail.com (L.F.S.M.); fortega@biol.unlp.edu.ar (F.O.); ymonroy@quimica.unlp.edu.ar (Y.M.); florencia.versino@ing.unlp.edu.ar (F.V.); loredeladino@quimica.unlp.edu.ar (L.D.); 2 Facultad de Ciencias Exactas, Universidad Nacional de La Plata (UNLP), 47 y 115, La Plata 1900, Argentina; 3 Facultad de Ingeniería, Universidad Nacional de La Plata (UNLP), 47 y 115, La Plata 1900, Argentina

**Keywords:** agro-industrial waste valorization, circular bioeconomy, non-traditional vegetal species, active and intelligent packaging, agronomic applications, bioactive compounds

## Abstract

This work reviews the strategic valorization of industrial by-products as sustainable sources of biopolymers and bioactive compounds, promoting a circular economy through the efficient use of renewable resources and reducing waste generation. These strategies contribute to lowering the carbon footprint of conventional packaging and plasticulture while supporting more resilient and diverse agriculture systems. Special emphasis is placed on processing roots and tubers as renewable raw materials for the production of biodegradable films for agronomic applications as eco-friendly alternatives to petroleum-based plastics and contributing to soil and ecosystem protection. Additionally, the incorporation of by-products from yerba mate (*Ilex paraguariensis*) demonstrate significant potential as both matrix-forming and filler materials in biodegradable composites while also providing antioxidant activity and pH-sensing capacity. This sustainable framework is further expanded through the utilization of non-traditional species like rosehip (*Rosa rubiginosa*), Aloe vera (*Aloe barbadensis)*, and topinambur (*Helianthus tuberosus*), which provide versatile functional matrices and bioactive compounds. Finally, the development of active and intelligent food packaging is addressed. Extracting natural pH-sensitive pigments from red cabbage and topinambur flowers enables the formulation of eco-friendly inks for real-time freshness monitoring. Ultimately, integrating these waste streams drives technological disruption, scaling sustainable, tailored solutions for global industry needs.

## 1. Introduction

The valorization of industrial by-products as a source of biopolymers and bioactive compounds represents a transformative strategy at the intersection of economic viability and environmental sustainability. By repurposing agricultural and processing residues, this approach mitigates the ecological burden of waste disposal and reduces reliance on fossil-based resources, directly aligned with the principles of the circular bioeconomy. Economically, it establishes new and low-cost value chains that convert financial liabilities, such as waste management costs, into high-value feed stocks. From a functional perspective, the extraction of polymers and compounds derived from biomass enables the development of innovative, biodegradable materials, including active food packaging that extends shelf life and sustainable agronomic materials, such as eco-friendly mulch films or controlled-release systems. Ultimately, this holistic utilization of by-products not only minimizes environmental footprints but also drives innovation across the agricultural and food packaging sectors.

Bibliometric analysis was conducted to highlight the main research topics addressed in the scientific literature and their thematic relationships. This methodology allows a quantitative approach to evaluate the scientific literature within a research field by analyzing publication patterns, citation relationships, and keyword co-occurrence, thereby identifying research trends, influential studies, and emerging topics.

**Figure 1:**
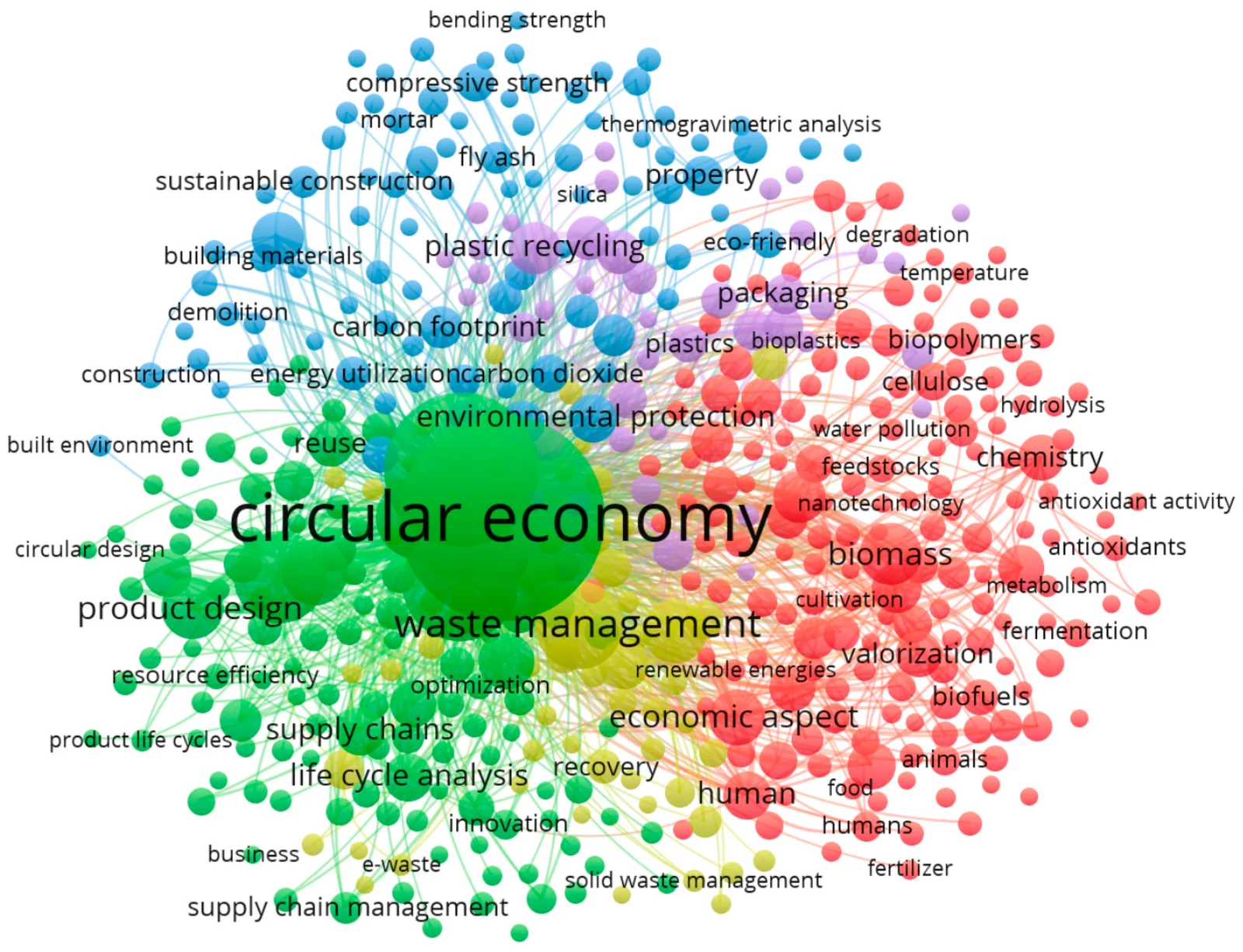
Keyword co-occurrence network map of publications from 2015 to 2025.

In this sense, a comprehensive search of the Scopus database was performed using key terms related to the “circular economy”, “sustainable materials”, and “by-products”. The search targeted publications from 2015 to 2025 in thematic areas including engineering, environmental and materials science, chemistry, pharmacology, toxicology and pharmaceutics, and agriculture. This search identified a total of 3423 documents, reflecting the extensive research activity focused on this field. Based on the information retrieved from the Scopus database, mapping was conducted through co-occurrence analyses using VOSviewer software (version 1.6.20). The minimum number of occurrences was set at 20, meaning that only keywords appearing at least 20 times in the dataset were considered for network construction. The keyword co-occurrence map (Figure 1) revealed five clusters, each represented by a specific color corresponding to a distinct keyword. The green cluster is centered on “circular economy”, the red cluster on “biomass”, the blue cluster on “construction industry”, the yellow cluster on “waste management”, and the purple cluster on “plastic recycling”.

The results show a sustained and progressive increase in interest in the topic, with a notable raise in contributions in 2025 (1219 documents), reflecting an increasing emphasis on sustainability and the circular economy. This trend highlights the emerging importance of biomass as a renewable resource for sustainable material development. Table 1 presents a reduced dataset version of the keywords shown in Figure 1, organized in ascending order of cluster number and including only keywords with Total Link Strength (TLS) > 2000. Total Link Strength facilitates the identification of key terms that play a central role in shaping and interconnecting the thematic structure of the field.

**Figure 2:**
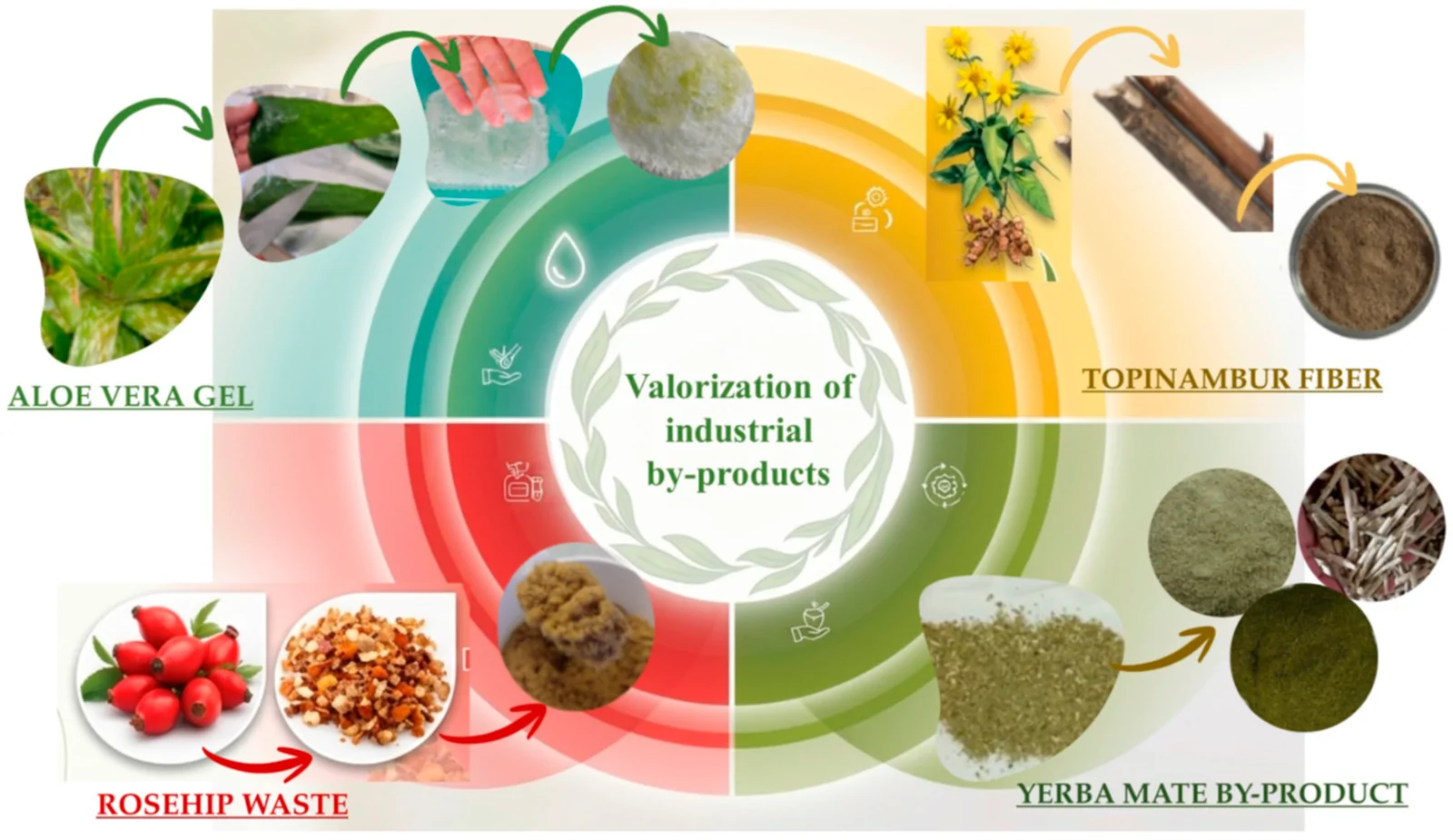
Valorization of non-conventional and industrial by-products.

In this context, guided by these emerging research trends, this study focuses on the valorization of residues derived from industrially relevant species as well as non-conventional biomass sources (Figure 2). Rather than limiting to traditional strategies that remain centered solely on the recovery of specific compounds, this work highlights the direct use of these residues as biomass resources that can contribute to the structural integrity of sustainable material formulations while simultaneously exploiting their bioactive constituents to impart functional properties. This integrated approach maximizes underutilized resource efficiency, which contributes to waste reduction, the development of sustainable materials and increasing the added value of agro-industrial by-products, supporting the transition toward circular production systems.

A previous study [1] has reported that agri-food waste, which encompasses a wide range of materials such as crop residues, animal manure, processing by-products, and post-consumer food waste, is often disposed of through landfills and open burning, leading to various environmental problems, including greenhouse gas emissions, soil and water pollution, and depletion of natural resources.

The composition of food waste varies depending on the specific source and geographic location. Plant-based waste is typically high in carbohydrates and fiber, with varying levels of lignin, cellulose, and hemicellulose. Food by-products and waste from various food industries are abundant sources of bioactive compounds, nutraceuticals, and naturally occurring substances beneficial to human health [2].

Proteins recovered from agricultural and food waste, such as wheat gluten, potato proteins, zein, soy, rapeseed, sunflower proteins, casein, whey proteins, blood-derived proteins, gelatin, collagen, keratin, and algal protein concentrates exhibit film-forming ability and functional properties. These proteins have been widely explored for the production of biomaterials intended for applications such as food packaging, agricultural materials, controlled-release systems, and tissue engineering, contributing to the valorization of agro-industrial waste within a circular bioeconomy framework [3]. As an example, zein represents promising building blocks for biopolymers and nanocomposites. For instance, D’Auria et al. [4] evaluated nanocellulose isolated from *Silybum marianum* as a sustainable functional filler in thermoplastic zein matrices, significantly improving their mechanical and moisture resistance properties for packaging applications. Similarly, the processability of thermoplastic zein blends through injection molding has been thoroughly studied [5].

Additionally, recent advances have focused on incorporating nanofillers into biomass-derived polymers to enhance their mechanical, barrier, and functional properties for food packaging applications [6,7]. In this context, potato starch-based biocomposites reinforced with cellulose nanofibers from potato peel waste have been successfully developed [8]. The synergistic effect of TiO_2_ and montmorillonite as dual reinforcements in potato starch nanocomposite films has also been shown to improve their thermal, mechanical, and barrier properties [9]. Recently, Nazar et al. [10] demonstrated that incorporating *Moringa oleifera* leaf extract loaded with *Coffea arabica*-derived carbon quantum dots enhances the functional performance of sweet potato starch nanocomposites.

The valorization of lignocellulosic residues and biomass resources in general constitutes a fundamental strategy for promoting sustainable development, with evidence showing that the effective utilization of such waste streams contributes meaningfully to the achievement of the Sustainable Development Goals (SDGs) [11]. Such approaches support responsible production and consumption patterns (SDG 12) and contribute to the generation of clean and renewable energy (SDG 7). In addition, the effective utilization of these wastes helps reduce environmental pressures across multiple dimensions, including water quality, urban sustainability, climate change, and terrestrial and aquatic ecosystems (SDGs 6, 11, 13, 14, and 15). More broadly, the integration of biomass and residue valorization strategies within industrial systems fosters technological innovation, infrastructure development, and economic growth (SDGs 8 and 9), reinforcing the transition toward more circular and resource-efficient bioeconomies [12].

Therefore, this review focuses on the valorization of agro-industrial by-products, emphasizing roots and tubers as renewable raw materials for eco-friendly agronomic films. Additionally, it examines yerba mate by-products as matrices, fillers, and functional additives providing antioxidant and pH-sensing properties. A distinctive aspect of this study is emphasizing non-traditional species—such as rosehip, Aloe vera and topinambur—for matrix development and as sources of bioactive compounds for active and intelligent food packaging.

## 2. Valorization of Highly Relevant Industrial By-Products

Plant-based agri-food side-streams (AFS) arise from agricultural production, harvest losses, and processing of crops, including cereals, sugar crops, fruits, vegetables, roots, tubers, oilseeds, pulses, and tree nuts. These residues, such as straw, husks, peels, pomace, and oil cakes, are generated in substantial quantities worldwide and represent a largely underutilized resource [13].

Biomass derived from food and feed crops serves as a critical source of high-value precursors, materials, and chemical constituents recoverable through waste valorization processes [14]. These resources are characterized by significant concentrations of cellulose, hemicellulose, lignin, and occasionally proteins, all of which may be repurposed to enhance food formulations. Currently, dietary fiber (DF) is recognized as a cornerstone of a healthy diet; consequently, its incorporation as a functional ingredient is highly promoted. Both soluble and insoluble fiber fractions exhibit a diverse array of techno-functional properties, including water-binding capacity, gelation, and fat replacement. Additionally, certain fiber-based products act as carriers for bioactive compounds [15]. This synergy has catalyzed the commercial availability of food products enriched with DF sourced from agricultural by-products. The global dietary fiber market was valued at USD 6.15 billion in 2025 and is expected to reach USD 10.63 billion by 2033, growing at a CAGR of 7.2% from 2026 to 2033 [16].

### 2.1. By-Products of Root and Tuber Processing

Potato *(Solanum tuberosum* L.) is one of the most widely cultivated and consumed food crops worldwide, with global production reaching approximately 376 million metric tons. The potato is among the most extensively cultivated and consumed food crops on a global scale, with production exhibiting a consistent upward trajectory to meet global food demands. Industrial processing of these products generates substantial quantities of waste materials, including peels, pomace, pulp, and process water. These by-products are frequently discarded without any commercial utilization, resulting in the loss of potentially valuable resources. However, these residues are notably rich in carbohydrates, proteins, and bioactive compounds, which makes them promising substrates for the extraction and bioconversion into a wide range of value-added products [12].

Cassava (*Manihot esculenta*) is a semi-woody shrub indigenous to tropical and subtropical regions. Its inherent resilience to drought and low soil fertility positions it as a strategic crop for climate change adaptation [17]. Global cassava production has consistently remained around 300–310 million tons per year in recent years, according to FAO statistics [18]. Roots are primarily processed into starch, flour, and chips, as critical feedstocks for the food, bioethanol, textile, and pharmaceutical industries. However, industrial processing generates significant environmental externalities; specifically, starch extraction produces ~11.1 kg of residue per 100 kg of product, often resulting in water pollution and odor issues [19].

Cassava bagasse, the primary industrial by-product, contains substantial levels of soluble and fibrous carbohydrates, making it a viable energy source for ruminant nutrition. Research has demonstrated favorable in vitro ruminal fermentation kinetics and robust microbial biomass development using this material [20]. Furthermore, the valorization of cassava bagasse into high-value bioenergy, such as bioethanol and bioelectricity, offers concurrent economic and environmental advantages [21].

In this context, ahipa (*Pachyrhizus ahipa*) emerges as a promising alternative starch source with physicochemical properties comparable to cassava. Native to the Andean regions of Bolivia and Peru, this *Fabaceae* species contributes to crop diversification and food security while mitigating the ecological risks associated with monocultures [22]. Although rich in starch (54–65% *w*/*w*, dry basis), ahipa roots are significantly more fibrous than cassava (87–91% db), leading to a substantially higher residue-to-starch ratio, approximately 1.09 kg of residue per kg of starch (db) [23].

To preclude potential environmental degradation during large-scale exploitation, it is essential to establish protocols for the valorization of these lignocellulosic residues. Polysaccharide properties, including solubility, swelling capacity, and digestibility, can be modulated through physical, chemical, or biological treatments [24]. Notably, physical modification methods are often prioritized, as they represent faster and safer alternatives to traditional chemical synthesis.

A previous study [25] investigated the main structural, physicochemical, and techno-functional properties of crude polysaccharides comprising the residues from ahipa and cassava starch extraction. The impact of thermal and ultrasonic treatments on the techno-functional and physicochemical properties of fibrous residues derived from ahipa and cassava starch extraction was evaluated. These physical modification techniques were selected as sustainable, cost-effective, and food-grade alternatives. While these processes facilitated starch removal, they significantly altered the structural integrity of the fibers, particularly in cassava, as evidenced by SEM, FTIR, and XRD analysis. Cassava residues exhibited substantial shifts in hydration properties (swelling powder, water binding, and water holding capacity), whereas ahipa residues remained structurally more resilient [25]. Notably, ahipa bagasse maintained significantly higher hydration capacities, oil binding capacity, and residual phenolic content than its cassava counterpart. Rheological characterization indicated that only cassava-derived aqueous suspensions exhibited weak gelling capacity. To enhance this functionality, a subsequent ultrasonic treatment was required; the resulting reduction in particle size and the increased proportion of carboxyl groups promoted the hydrophobic interactions necessary for gel stabilization. Given their distinct techno-functional profiles, treated cassava residues are suitable for improving texture in low-calorie spreads, while ahipa residues are better positioned for fiber enrichment in bakery products [25]. A similar trend was reported by Monroy et al. [26], who showed that ultrasonic treatment of cassava starch induces pronounced structural disruption and microstructural modifications, mainly evidenced by changes in granule morphology and crystallinity. These changes were further validated through a combination of complementary analytical techniques, including ATR-FTIR spectroscopy, microscopic analyses (SEM and CLSM), XRD, and thermal characterization, confirming the extent of molecular and structural reorganization resulting from the physical treatment. In summary, the results demonstrate that a range of starch derivatives with distinct characteristics can be obtained by tailoring ultrasound processing conditions, with distinct structural and functional characteristics, facilitating their targeted application across a wide range of industrial sectors.

**Figure 3:**
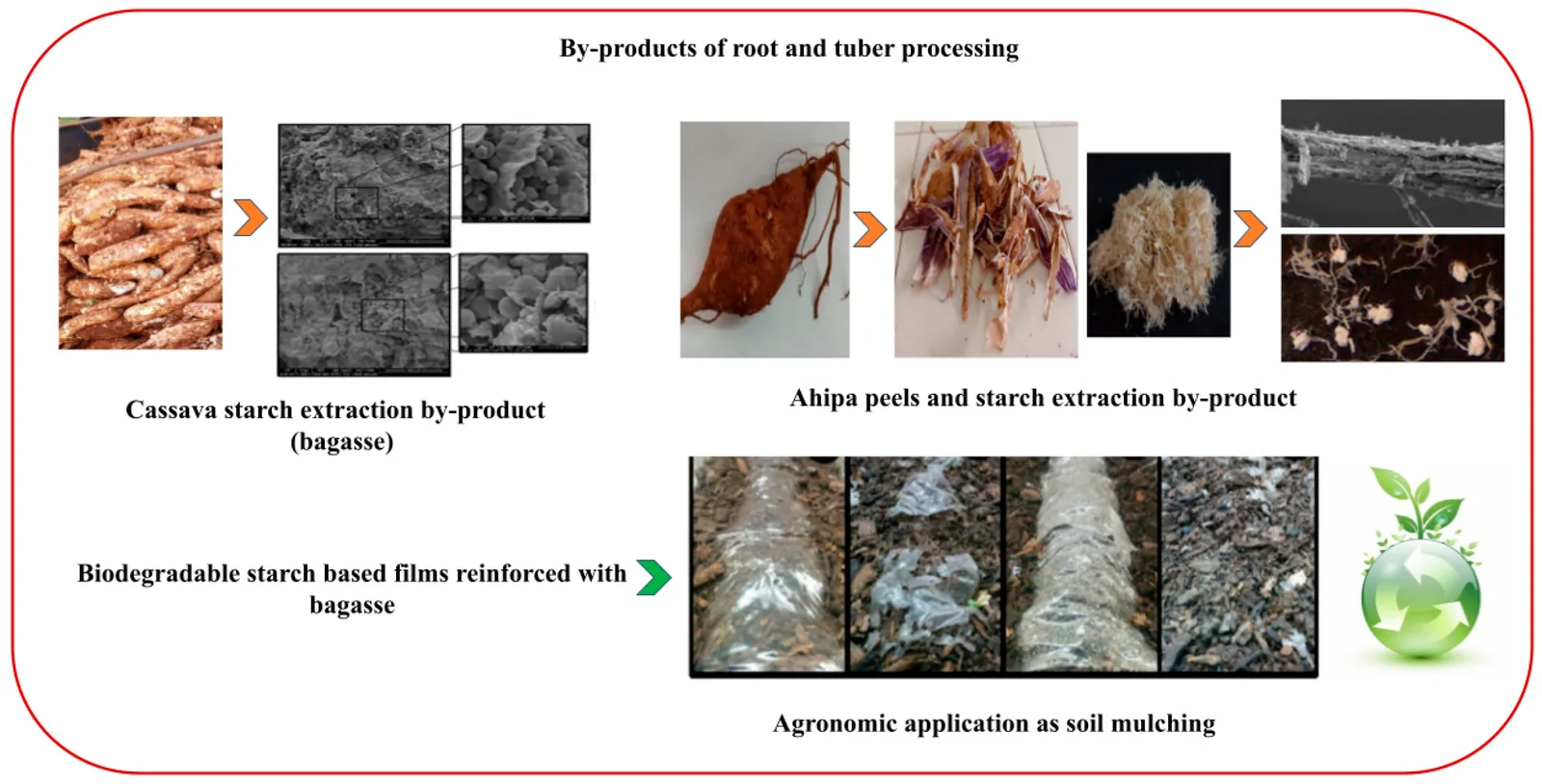
By-products of cassava and ahipa starch extraction processing and their application in the development of biodegradable films for agronomic applications.

Versino et al. [27] developed and characterized biodegradable, eco-compatible materials derived from cassava starch reinforced with natural fibers from bagasse and root peel, evaluating their mechanical, optical, and barrier properties. The study also examined the effects of various plasticizers and additives used to functionalize the matrix, alongside different processing techniques. The potential application of these materials in specific agronomic contexts, namely soil mulch and controlled-release fertilizer systems for seedling production, was assessed. To this end, the stability of the developed materials under diverse storage conditions, their soil biodegradation rates, and their varying modes of application were evaluated (Figure 3).

### 2.2. Yerba Mate Industrial By-Products

Yerba mate (*Ilex paraguariensis*) is an evergreen tree and industrial crop from the Aquifoliaceae family native to South America, particularly Argentina, Uruguay, Paraguay, and Brazil. Its processed leaves are widely consumed as hot and cold infusions, including chimarrão, tereré, and mate tea, and it is commercially cultivated, produced, and industrialized mainly in Northeastern Argentina, Paraguay, and Southern Brazil, reaching an annual production of thousands of tons. Argentina is the largest producer and consumer of yerba mate, with 266.788,512 t of mill output destined for the domestic market during 2025, and 57.980,912 t intended for the international market [28]. During yerba mate processing, various products are obtained, such as leaves, stems, and powder (Figure 2) [29].

Driven by increasing interest in the valorization of agro-industrial residues, the number of studies focusing on yerba mate by-products has increased considerably over the last decade. According to the Scopus database, research output remained limited between 2016 and 2022, with no more than two publications per year. However, publication activity has grown markedly since 2023, reaching eight documents in June 2026. This trend reflects the increasing scientific interest in exploring yerba mate by-products as sustainable sources of valuable compounds and raw materials for food, pharmaceutical, and biomaterial applications. Regarding the geographical distribution of the publications reported in Scopus, it is noteworthy that, while the majority of research originates from Argentina and Brazil (the leading yerba mate-producing countries), growing interest in this subject is reflected in contributions from more distant regions, including Italy, New Zealand, Canada, Turkey, and Vietnam, suggesting that yerba mate by-products are gaining international scientific attention.

In line with the growing interest in the valorization of agro-industrial residues, studies focused on bioactive compounds from yerba mate by-products have also increased markedly in recent years, particularly since 2023, reflecting the potential of these materials as sources of high-value functional ingredients [30,31]. This trend may be attributed to the high concentration of bioactive compounds present in these by-products, particularly polyphenols, which are associated with antioxidant activity and several beneficial effects on human health [32,33,34].

With respect to the use of yerba mate for the development of films, either as a matrix-forming compound, filler, or active component, out of a total of 19 articles reviewed in Scopus, 4 used household yerba mate waste [35,36,37,38] and one used the agronomic residue from the plant’s fruits, while the remainder used extracts from commercial packages [39,40,41,42,43,44,45], dried yerba mate leaves [46,47], or fruits [48,49].

**Figure 4:**
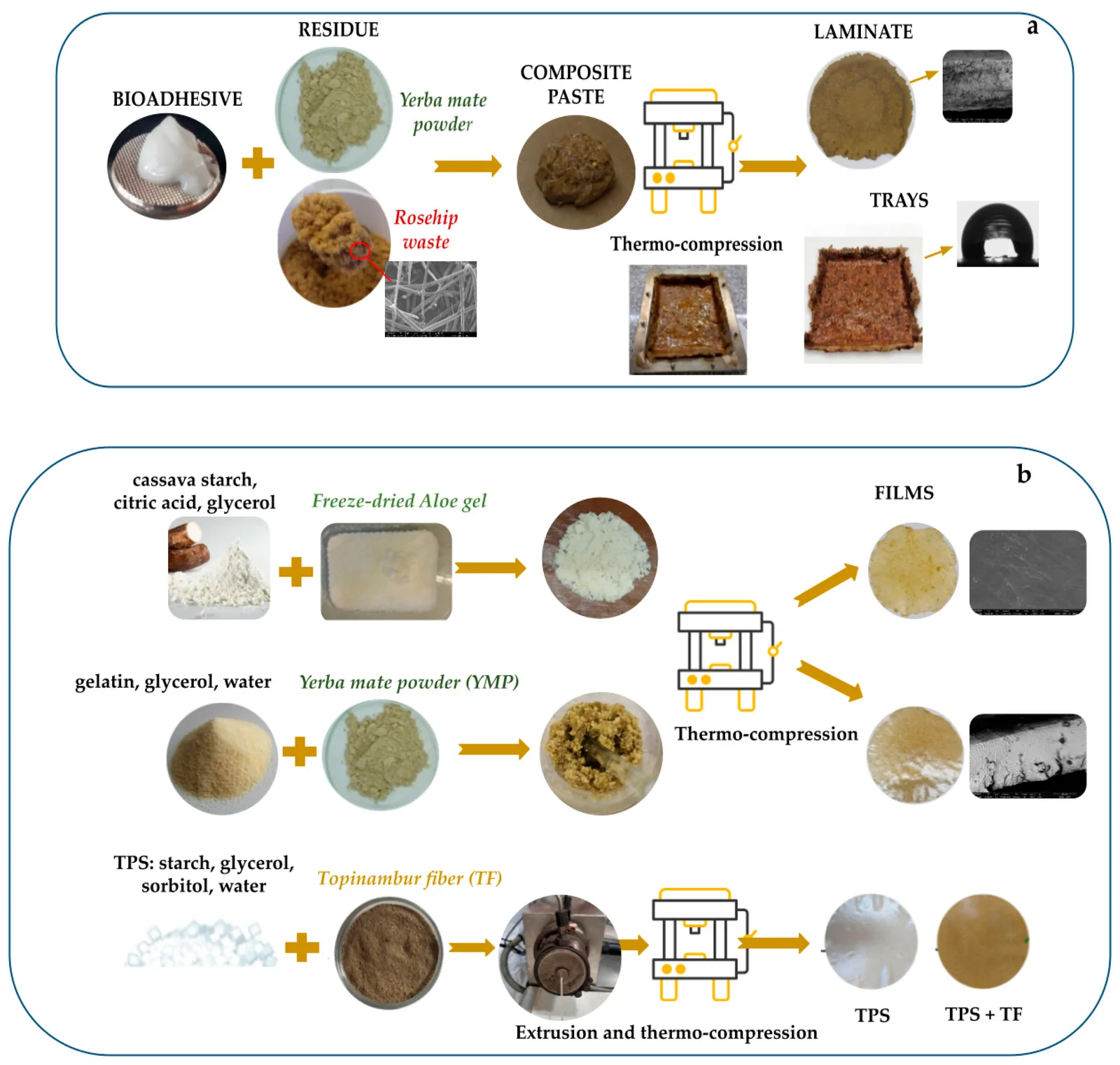
Revalorization of agro-industrial by-products as reinforcement in materials obtained by thermo-compression and extrusion: (**a**) laminated materials or trays and (**b**) active biodegradable films.

Notably, studies employing by-products in their native, untreated form remain scarce [50,51]. Therefore, investigating their direct utilization constitutes an attractive strategy for enhancing the value of these materials while minimizing additional processing steps (Figure 4a,b).

In this context, the use of yerba mate powder (YMP) as a lignocellulosic source represents an innovative approach for the design of biopolymeric matrices. Therefore, Monroy et al. [50] determined the lignin content of YMP (5.5%), hemicellulose (8.9%), and cellulose (21.4%). They capitalized on this biomass to develop biodegradable mono- and bilayer tray-like materials via thermo-compression, using bioadhesive formulations based on chemically modified cassava starch as an eco-friendly and sustainable alternative (Figure 4a).

Furthermore, raw YMP plays a dual role in the manufacturing of films and/or composite materials. Given its particle size (250–700 μm) [52], it can act as a micrometric filler. Furthermore, the presence of phenolic acids, flavonoids, methylxanthines, and saponins contributes to the high bioactive potential of YMP, conferring antioxidant, anti-inflammatory, hypolipidemic, and neurostimulant properties [53].

Taking advantage of this dual role, Laszeski et al. [51] developed a composite material based on gelatin and YMP, using glycerol and water as plasticizers, thereby enabling biopolymer processing through thermo-compression (Figure 4b). Accordingly, the presence of YMP imparted several functional properties to the resulting films. Controlled antioxidant release was achieved, as these compounds modified the microstructure of the gelatin matrix, exerting a twofold effect by acting as plasticizers or crosslinking agents depending on their concentration. In addition, the films exhibited strong UV-blocking properties, making them a promising option for protecting photosensitive systems. The material also demonstrated pH responsiveness by sensing alkaline conditions, suggesting potential applications in shelf-life extension and spoilage monitoring.

Another application of this by-product has been the production of biofertilizers. YMP is 90% organic matter, and the 10% ash is composed of K, Fe, Mg, Mn, Cu, and Na, secondary nutrients and micronutrients essential for soil fertilization [54]. Based on these properties, Ca(II)-alginate-based capsule matrices with final YMP contents of 50–80% *w*/*w* were developed [52,54]. These fertilizer systems exhibited low hygroscopicity, high crushing strength, and elevated encapsulation efficiencies for urea and phosphorus while also showing biodegradability, complete nutrient release in soil, and no inhibitory effects on soil microorganism growth. Moreover, their simple and low-cost production process, together with the efficient use of unmodified agricultural residues, highlights their potential for large-scale fertilizer manufacturing. Similarly, residues generated after brewing yerba mate were shown to retain significant elemental richness, despite not being agro-industrial by-products [55]. Through a three-stage elemental assessment strategy, these residues were proposed for reuse as natural fertilizer, offering a sustainable alternative for waste management.

## 3. Sustainable Processing of Underutilized Vegetal Species: A By-Product Utilization Approach

The growing demand for environmentally sustainable materials has driven increasing interest in the development of bioplastics, biomaterials, and biocomposites derived from agro-industrial residues such as husks, stalks, peels, and shells. In this context, the transition toward a fossil-free economy is being reinforced by the valorization of agricultural waste streams, particularly lignocellulosic resources such as stalks and bark, which serve as renewable feedstocks to produce eco-friendly materials. This approach not only contributes to waste reduction and environmental pollution mitigation but also aligns with the expanding market demand for sustainable and responsibly sourced alternatives, thereby supporting the broader implementation of circular bioeconomy strategies [56].

Beyond traditional agricultural residues, non-conventional species such as Aloe vera and rosehip generate a considerable amount of by-products during processing that, despite their enormous biotechnological potential, are usually relegated to the background compared to more common waste such as sugarcane bagasse or corn stubble.

### 3.1. Aloe Vera

Aloe vera (*Aloe barbadensis*) is a perennial succulent plant of the Asphodelaceae family that grows in arid and warm tropical regions worldwide. Its thick leaves contain a transparent gel composed of more than 98% water and small amounts of carbohydrates, dietary fiber, amino acids, proteins, vitamins, minerals, organic acids, and other bioactive compounds [57]. Owing to its physicochemical properties, Aloe vera gel exhibits excellent compatibility with different biopolymers, making it an attractive component for biodegradable films and edible coatings [57,58].

Although the gel is the primary commercial product, Aloe vera has attracted increasing attention as a renewable biomass resource because its industrial processing generates considerable amounts of underutilized leaf residues. These by-products remain rich in polysaccharides such as acemannan and fructans, as well as phenolic compounds, flavonoids, anthraquinones, chromones, vitamins, and other bioactive constituents with antioxidant and antimicrobial properties [59,60,61]. Consequently, Aloe vera waste has been explored as a feedstock for functional food ingredients, biosorbents, biofuels, natural polymers, and biomedical applications, including drug delivery systems, supporting its valorization within a circular bioeconomy framework [55,56,57,58,59,60,61].

The film-forming capacity of Aloe vera gel, together with its antimicrobial and antioxidant activities, has promoted its use as a natural alternative to synthetic preservatives in food packaging [62]. For example, carrageenan-based films containing Aloe vera gel showed antibacterial activity against Escherichia coli and reduced lipid oxidation in cheese [63]. Likewise, Aloe vera-based edible coatings have been successfully applied to fruits and vegetables, where they act as semipermeable barriers to water vapor and gases, delaying ripening, preserving quality attributes, and extending shelf life [63,64,65]. These properties have positioned Aloe vera as a multifunctional ingredient for food preservation and sustainable packaging applications.

While previous reviews have comprehensively addressed the phytochemical composition, biological activities, processing technologies, and food applications of Aloe vera [57,60,63], its role as a biomass resource for sustainable material development and waste valorization has received comparatively less attention. Moreover, aspects related to formulation strategies, regulatory considerations, and the translation of laboratory-scale developments into food-grade applications are often discussed separately rather than within an integrated framework. This highlights the need for a broader perspective on the utilization of Aloe vera and its by-products in the development of sustainable materials and packaging systems.

### 3.2. Rosehip

*Rosa rubiginosa* L. (sweet briar or *rosa mosqueta*) is a widely distributed species, native to parts of Europe, Asia, North America, and North Africa, and considered invasive in regions such as Australia, New Zealand, and South America. Its processing, particularly for oil extraction, generates substantial quantities of residues, such as husk, seed cake, and “fluff”. These by-products remain largely underutilized despite their potential within circular bioeconomy strategies (sometimes used as low-value fertilizer or converted into briquettes for heating) [65]. These residues are rich in lignocellulosic components (cellulose, hemicellulose, and lignin), as well as residual lipids and phenolic compounds, making them attractive feedstocks for bio-based materials [66]. Despite its abundance, there is still limited information on large-scale production and, more importantly, relatively few studies have explored its potential revalorization as a biomass resource. While some research has begun to investigate its use within a biorefinery framework for obtaining biopolymers and biofuels, these approaches remain scarce, highlighting opportunities for developing high-value, zero-waste applications [67].

Among these residues, the fluff generated during oil extraction has a lignocellulosic matrix ideal for developing rigid or semi-rigid biomaterials (trays and laminates) [65] (Figure 3). Cellulose microfibrils, characterized by their highly crystalline structure, act as the primary reinforcing or filler elements within the biopolymer matrix, largely determining the mechanical strength, stiffness, tensile resistance, and thermal stability of the resulting material [65]. Meanwhile, hemicellulose, an amorphous and branched polysaccharide, promotes cohesion, contributing to flexibility and interfacial compatibility and processability [66]. Lignin contributes to stiffness, moisture resistance, and intrinsic UV-shielding and antioxidant activity properties, making this a promising reinforcement for biodegradable food packaging materials [65,67].

### 3.3. Topinambur

Topinambur (*Helianthus tuberosus*), also known as Jerusalem artichoke, a plant indigenous to North America, exhibits remarkable resilience to diverse environmental conditions, requiring minimal fertilizers, pesticides, and irrigation, factors that have been pivotal in its global expansion. This crop has attracted significant agro-industrial interest primarily due to its tubers, which are a rich in inulin and fructooligosaccharides, widely used in functional and nutraceutical products for diabetic and celiac populations, and bioenergy applications [68,69]. In contrast, its aerial biomass, which can reach heights of 3 m, remains largely underutilized despite a substantial lignocellulosic fraction. The valorization of this renewable feedstock for bio-based composites represents an attractive strategy within the circular bioeconomy, creating value from agricultural residues while reducing environmental impacts.

Sierra Montes et al. [70] characterized the lignocellulosic residues derived from topinambur cultivation. These fibers contain around 40% cellulose, 14% hemicellulose, and 24% lignin. The authors proposed the integral valorization of these fibers as reinforcing agents in thermoplastic cassava starch (TPS) films (Figure 4b). Unlike conventional fiber modification approaches that rely on chemical delignification or other energy-intensive treatments that rely on energy-intensive and hazardous chemical treatments [71], untreated fibers were successfully incorporated into TPS films by extrusion and thermo-compression, using conventional polymer-processing equipment, highlighting the scalability of this approach.

The incorporation of topinambur fibers improved the UV barrier properties, tensile strength, elastic modulus, and moisture resistance of TPS films while maintaining homogeneous filler dispersion and preserving biodegradability [70,72]. These enhanced properties make the resulting biocomposites suitable for agroecological applications, including biodegradable seedling pots and agricultural films that can be directly incorporated into soil after use.

Overall, by integrating agricultural residues into low-cost, eco-friendly, high-performance biodegradable agro-films, a sustainable field-film-field (F-F-F) closed-loop strategy is established that supports soil conservation, reduces plastic waste, and promotes circular resource utilization.

## 4. Unconventional Usual Industry By-Products for Advanced Packaging Solutions: Active and Intelligent Packaging

Circular economy strategies are redefining the global landscape by creating new opportunities to transform waste into value-added products, energy, and functional materials. In this context, significant amounts of vegetable waste are generated by food processing industries and households, including peels, seeds, and pomace, whose improper disposal poses serious environmental, economic, and food security challenges [57]. However, these horticultural by-products contain different biomolecules, such as anthocyanins, organic acids, phenolic compounds, and fibers, that can be used as high-value functional ingredients [56]. These by-products represent renewable raw materials for biodegradable matrices and can impart sensing, antioxidant, and antimicrobial functionalities, enabling the design of multifunctional packaging with built-in responsiveness. In this context, active packaging systems are designed to interact with food or its surrounding environment to extend the shelf life, while intelligent packaging systems provide real-time information about food quality through integrated sensing mechanisms [73].

Among the different types of biomolecules, natural pigments, especially polyphenols and anthocyanins, have received increasing attention due to their pH-sensitive color transitions and intrinsic antioxidant properties, allowing them to act simultaneously as freshness indicators and active compounds in food packaging applications [74]. Anthocyanins are found in flowers, leaves, fruits, stems, and roots of a variety of plants, including blackberries, black carrots, red cabbage, beets, sweet potatoes, and topinambur, among others. In line with this, recent studies have demonstrated the successful incorporation of horticultural extracts into biodegradable matrices to develop dual-function materials. For example, sericin-based films with red cabbage extracts have demonstrated the capacity to extend food shelf-life and provide real-time visual freshness monitoring, highlighting the feasibility of integrating active and intelligent functionalities within a single system [75]. In addition, yerba mate (*Ilex paraguariensis*) is a plant rich in polyphenols, primarily flavonoids and xanthines, with antioxidant and anti-inflammatory properties. Recently, films based on cassava starch, pea flour, and yerba mate extract were developed [69]. These films exhibited a color change under alkaline conditions, particularly at pH 6.5 and above. Their application to fish preservation confirmed their dual function. The films acted as real-time indicators of fish quality, showing visible color changes from light brown to yellowish brown over 12 days, while also extending the shelf life of packaged fish and maintaining it safe for consumption for up to 12 days at 4 °C. In this regard, intelligent biopolymer systems using yerba mate waste as functional agents were also proposed [76].

**Figure 5:**
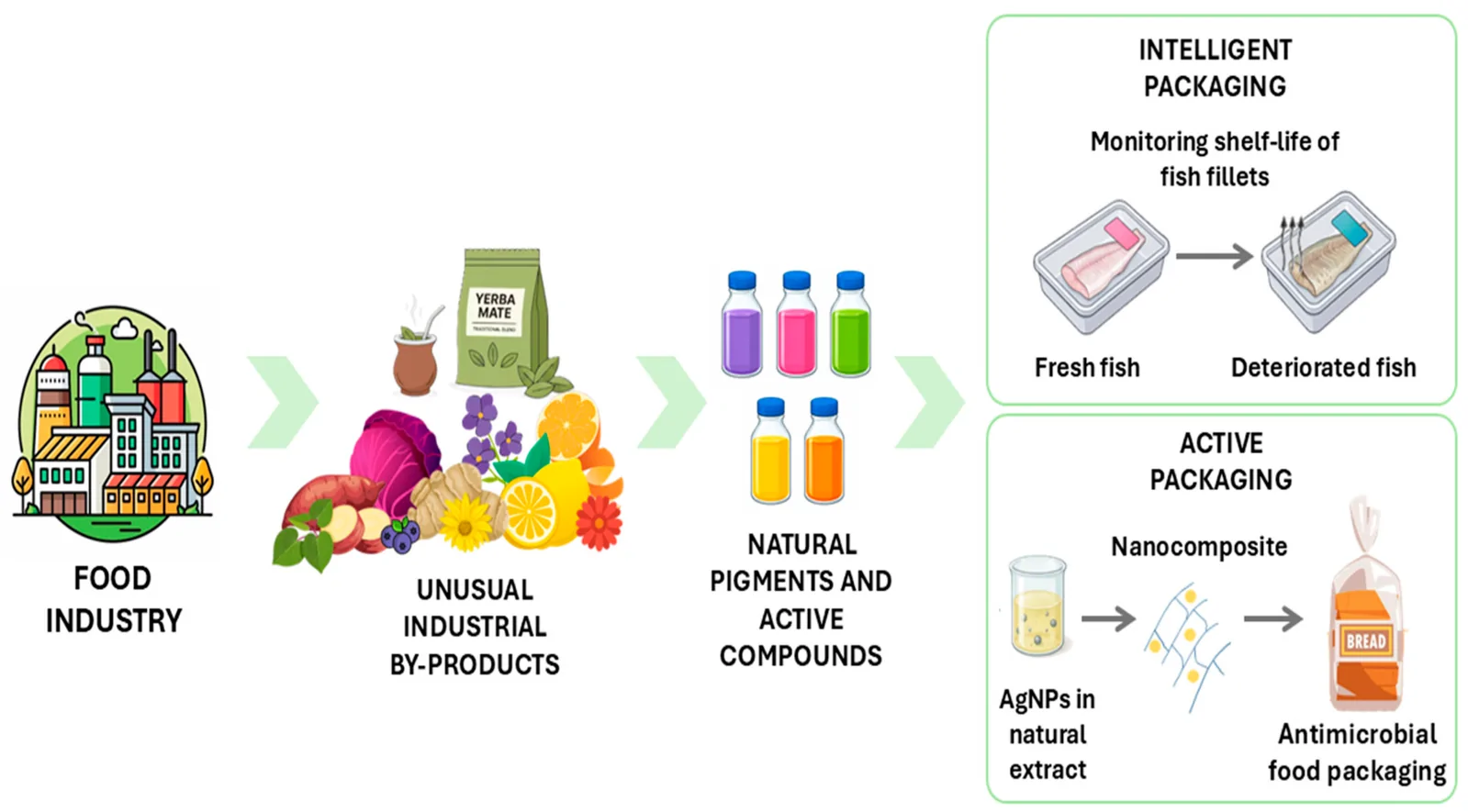
Valorization of horticultural waste for the development of intelligent materials and their application in food shelf-life monitoring.

Additionally, in recent years, the valorization of flowers and their extracts has gained significant attention as a sustainable source of potent bioactive compounds, such as polyphenols, flavonoids, and anthocyanins. These natural extracts possess remarkable antioxidant and antimicrobial properties, making them excellent candidates for the development of active food packaging that actively extends the shelf life and preserves the quality of perishable goods. Due to the pH-sensitive color-changing properties of certain floral pigments (particularly anthocyanins), these extracts are being innovatively integrated into intelligent packaging systems. These smart materials can visually monitor and communicate food freshness or spoilage in real time, offering a non-destructive, eco-friendly, and consumer-friendly alternative to conventional synthetic packaging (Figure 5). Dobrucka et al. [77] developed polysaccharide-based films incorporated with *Cannabis sativa* flower extract to extend the shelf life of freeze-dried fruits, such as raspberries and blueberries, through their robust antimicrobial and antioxidant properties. In this sense, the flowers of topinambur, which are generally discarded, have been reported to contain a variety of bioactive compounds, including aurones, chalcones, flavones, flavonols, coumarins, and chromones, which possess conjugated structures that are sensitive to changes in their chemical environment [78,79]. The presence of these compounds, together with their antioxidant and antimicrobial properties, suggests that these flowers could be used as an alternative source of sensing and active molecules in the development of multifunctional packaging systems.

According to Bertolo et al. [80], citrus by-products (peels, pomace and seeds) have been used as raw materials in food packaging film formulation due to their composition. These by-products contain pectin, which can be used as a polymeric matrix, and their bioactive compounds (phenolic acids, flavonoids, carotenoids, essential oils, among others) can be added to this polymeric matrix to improve its functionality and physical properties, allowing for intelligent and active food packaging. Among these bioactive constituents, carotenoids, which are fat-soluble pigments, require particular attention as active rather than as sensing compounds [81]. Although they exhibit pH-dependent optical changes, they are less sensitive than anthocyanins, which limits their use as colorimetric indicators. Carotenoids are valued for their antioxidant activity, contributing to food preservation and extending shelf life. Additionally, their ability to absorb ultraviolet radiation improves the light barrier properties of packaging materials, protecting foods from oxidative degradation. These multifunctional attributes make carotenoid-rich horticultural by-products attractive for developing active packaging systems [82]. In addition to being directly incorporated into polymeric matrices, these horticultural by-products can play an active role in the green synthesis of functional nanomaterials. For instance, lemon juice has been used as a reducing and stabilizing agent to synthesize silver nanoparticles, which were then incorporated into starch-based nanocomposite films [83]. This eliminates hazardous reagents from the synthesis process and enhances the antimicrobial activity and functional properties of the resulting packaging material.

Thus, using horticultural by-products as a source of structural biopolymers and functional compounds is a promising strategy for developing next-generation sustainable, multifunctional, real-time responsive packaging systems.

## 5. Feasibility Assessment and Future Perspectives

Current research paradigms have pivoted toward the development of eco-compatible starch-based matrices reinforced with fibrous fillers derived from agro-industrial and food residues, including sugarcane bagasse, corn husks, yerba mate powder, and malt bagasse [26,73,84]. The valorization of these lignocellulosic by-products represents a strategic approach to mitigating the escalating pollution crisis while enhancing the added value of such resources. This shift yields substantial ecological benefits, particularly regarding waste minimization and the reduction of the overall carbon footprint. However, the large-scale implementation of these materials depends not only on their environmental performance but also on overcoming technical, regulatory, and logistical challenges that currently limit their commercialization.

To ascertain the techno-economic viability of food waste valorization, a rigorous cost–benefit analysis is indispensable. This evaluation ensures that the environmental advantages of waste valorization are balanced with economic feasibility, processing efficiency, and scalability. The existing literature indicates that despite the significant promise of food-waste-based bioprocesses, their transition to commercial scale is predicated upon robust financial performance and technological optimization. In this sense, Patel et al. [85] proposed integrated biorefineries to valorize food waste through biological and thermochemical processes, producing biofuels, nutraceuticals, and biomaterials. Their study highlights the importance of economic analysis to drive the circular economy and mitigate environmental impact. Beyond economic considerations, commercialization is further challenged by the intrinsic variability of biomass feedstocks, whose composition depends on species, cultivation conditions, geographical origin, and seasonal availability, affecting process reproducibility and product performance.

Regulatory and standardization bottlenecks also represent major barriers to market adoption. Existing technical standards, certification schemes, and safety regulations have largely been developed for petroleum-based materials and often fail to adequately address the characteristics of emerging bio-based alternatives. For example, the European Union Packaging and Packaging Waste Regulation (PPWR) establishes increasingly stringent requirements for packaging recyclability, recycled content, compostability, and extended producer responsibility, creating both opportunities and compliance challenges for novel bio-based materials [86]. In the United States, market access depends on compliance with FDA food-contact regulations, USDA BioPreferred requirements for bio-based content, and ASTM standards (e.g., ASTM D6400, D6868, and D6866) [87,88,89] that underpin compostability certification and are incorporated into regulations in several states, including California and Washington.

The absence of harmonized methodologies for assessing biodegradability, compostability, durability, and long-term performance, together with fragmented biomass supply chains, limited industrial infrastructure, and insufficient technical knowledge among manufacturers and end users, continues to hinder large-scale commercialization. These barriers are exacerbated by lengthy and costly certification processes that hinder the commercialization of innovative bio-based materials. Addressing these challenges will require coordinated efforts among researchers, industry, and policymakers to establish robust regulatory frameworks, resilient supply chains, and standardized performance criteria that facilitate the transition toward a circular bioeconomy.

Furthermore, Life Cycle Assessment (LCA) is a valuable tool for assessing the impact of sustainable materials beyond financial considerations. Bishop et al. [90] provided an extensive review on how LCA methodology helps compare and decide upon the environmental impacts of bioplastics relative to their petrochemical counterparts. An effective LCA for green biocomposites must account for the agricultural impacts of bio-based compounds, particularly eutrophication, pesticide and fertilizer use, and soil and water degradation. Furthermore, replacing commodity polymers with biodegradable matrices or incorporating natural fillers frequently requires physical or chemical modifications to achieve performance comparable to conventional materials. However, these processing steps incur additional production costs and may not always align with eco-friendly standards.

Finally, artificial intelligence (AI) is recognized as a powerful driver of the circular economy, enabling production systems to improve resource efficiency, reduce waste generation, and strengthen their alignment with sustainability goals. In this context, AI acts as a disruptive catalyst in material science, completely redefining the engineering of sustainable, biodegradable solutions through the intelligent transformation of agro-industrial waste into high-value materials tailored to specific industrial applications [91]. Recent advances in machine learning and polymer informatics demonstrate that AI can accelerate the discovery of bio-based materials by predicting structure–property relationships, identifying optimal formulations and reducing the need for extensive experimental screening. AI-driven models are increasingly used to optimize processing conditions for extrusion, compression molding, and additive manufacturing, while digital twins and predictive algorithms enable real-time process control, improving product quality, reducing energy consumption, and minimizing material waste. Furthermore, AI can assist in predicting biomass availability, managing supply chain variability, and integrating life cycle assessment into material design, allowing environmental, technical, and economic criteria to be optimized simultaneously. AI has the potential to enhance packaging sustainability by decreasing waste, reducing emissions, and optimizing resource usage. At the same time, high costs, technical challenges, and regulatory issues continue to slow adoption. Emerging solutions, such as interdisciplinary collaboration, model enhancement, and consumer-centric design, offer ways to address these barriers [92].

Likewise, the integration of artificial intelligence into food packaging technologies is expected to transform the design, production and performance evaluation of next-generation packaging systems. AI-driven approaches can accelerate the development of active and intelligent materials, improve the monitoring of food quality, enhance safety management and optimize industrial processes. However, overcoming challenges related to data availability, regulatory harmonization, implementation costs, model transparency, and consumer acceptance will be essential for large-scale deployment. Future research should emphasize integrating sustainable materials, biodegradable sensing platforms, predictive modelling tools and digitalized manufacturing strategies. These advances could pave the way for highly efficient, intelligent and environmentally sustainable packaging solutions that align with the principles of the circular economy [93].

## 6. Conclusions

This review highlights the significant potential of agro-industrial residues and underutilized by-products derived from both widely cultivated and non-conventional plant species as renewable feedstock for the development of sustainable bio-based materials. By examining diverse biomass sources, including yerba mate, rosehip, Aloe vera and topinambur, it demonstrates that these resources can serve as sources of both structural components and bioactive compounds, such as polyphenols and pigments with antioxidant and antimicrobial capacities. This dual potential positions these resources as strategic feedstocks within circular bioeconomy frameworks aimed at generating high-value, sustainable alternatives to conventional fossil-based products.

The studies discussed throughout this review demonstrate that biomass-derived materials present remarkable versatility for tailor-made design materials in applications spanning from active and intelligent food packaging to agronomic mulch films, seedling pots, or controlled-release fertilizer systems. Its processability through scalable techniques like thermo-compression and extrusion highlights their compatibility with existing industrial infrastructure, facilitating the transition from laboratory-scale development to commercial implementation. The incorporation of renewable resources into biodegradable materials contributes to reducing dependence on fossil-based plastics, improving resource efficiency, and promoting more sustainable production systems. Furthermore, the development of active and intelligent packaging based on naturally derived bioactive compounds offers promising opportunities to enhance food quality and safety, reduce food losses, and contribute to the Sustainable Development Goals.

Despite these advances, several challenges remain before these technologies can achieve widespread industrial implementation. Future research should prioritize improving the consistency and performance of biomass-derived materials, developing standardized methodologies for evaluating biodegradability and long-term functionality, and establishing harmonized regulatory frameworks that facilitate commercialization. Strengthening biomass supply chains, reducing certification costs, and improving consumer confidence will also be essential for accelerating market adoption. In parallel, recent advances in AI, polymer informatics, and digital manufacturing are expected to play a crucial role in the next generation of active and intelligent packaging materials. Beyond its current application in process optimization, these technologies are driving a paradigm shift towards “tailor-made materials design” approaches, where desired functionalities, such as antioxidant and antimicrobial activity, gas barrier properties, mechanical performance, biodegradability under composting conditions, or controlled release, can be predefined based on the composition of the starting system and used as input parameters for predictive models. Therefore, the convergence of biomass valorization, advanced manufacturing, and data-driven technologies is expected to play a pivotal role in the development of next-generation sustainable materials capable of addressing global environmental and societal challenges while supporting the transition toward a resilient and circular bioeconomy.

## Figures and Tables

**Table 1:** Most frequent keywords and their co-occurrence metrics in publications from 2015 to 2025 *.

Keyword	Cluster Number	Links	Total Links Strength (TLS)	Occurrence	Average Publication Year
biomass	1	414	2882	237	2023.1857
chemistry	1	363	2131	133	2023.4286
economic aspect	1	430	3635	199	2022.7688
human	1	401	2586	153	2022.549
nonhuman	1	389	2616	137	2023.0365
circular economy	2	477	20,371	2636	2023.0493
economics	2	420	2365	205	2021.9659
environmental impact	2	468	5248	425	2022.9388
industrial economics	2	403	2170	206	2023.2767
life cycle	2	451	6224	522	2022.5536
life cycle analysis	2	376	2265	148	2022.2365
life cycle assessment	2	436	3675	279	2023.2688
product design	2	369	2447	249	2022.3012
recycling	2	475	8068	704	2022.5568
sustainability	2	475	7561	845	2022.6911
sustainable development	2	477	15,197	1462	2022.8666
waste disposal	4	414	2399	159	2022.3459
waste management	4	470	6036	497	2022.8692
plastic recycling	5	339	2006	173	2023.341

* Cluster number refers to the group to which each keyword belongs in the co-occurrence network. Links indicate the number of direct connections between a keyword and others. Total Links Strength (TLS) reflects the overall strength of these connections. Occurrence is the number of times the keyword appears in the dataset. Average publication year represents the mean year of publication of the documents in which the keyword appears.

## Data Availability

No new data were created or analyzed in this study. Data sharing is not applicable to this article. A comprehensive literature search was conducted using the Scopus database, covering publications from January 2015 to December 2025. The bibliographic dataset was retrieved through an advanced search strategy that combined author-defined search terms (TITLE-ABS-KEY) with the Scopus EXACTKEYWORD field. The initial search terms were selected according to the scope of the review and included combinations of the following keywords: “circular economy”, “sustainable material”, “by-product", while EXACTKEYWORD terms were incorporated to capture standardized indexed concepts and improve the completeness and reproducibility of the search. These were limited to: “Circular Economy”, “Sustainability”, “Biomass”, “Valorization”, “Food Waste”, “Waste”, “Chemistry”, “Waste Disposal”, “Agricultural Wastes”, “Wastes”, “Packaging Materials”, “Sustainable Materials, “Waste Valorization”, “Packaging”, “Waste Valorizations”, “Polymer”, “Cellulose”, “Industrial Waste”, “Waste Utilization”, “Bio-based”, “Extraction”, “Bioeconomy”, “Biopolymers”, “Lignin”, “Food Packaging”, “Sustainable Production”, “Biodegradation”, “Eco-friendly”, “Waste Products”, “Composite Materials”, “Industrial By-products”, “Fertilizers”, “Byproduct”, “Bioactive Compounds”, “Biodegradability”, “Solid Wastes”, “Sustainable Development Goals”, “Circular Bioeconomy”, “Solid Waste”, “Green Chemistry”, “Value Added Products”. The search was limited to research articles, review articles, conference papers, books, and book chapters. The Boolean operator “AND” was used to retrieve records containing all the predefined search concepts, thereby refining the search to the intersection of the selected terms. Exact phrases were searched using quotation marks where appropriate, ensuring precise retrieval of the intended terms. Microsoft Office Excel software was used to analyze the data, and VOSviewer was used to create the keyword co-occurrence network map.

## Abbreviations

The following abbreviations are used in this manuscript:

TLS	Total link strength
SDGs	Sustainable Development Goals
AFS	Agri-food side streams
DF	Dietary fiber
SEM	Scanning electron microscopy
FTIR	Fourier transform infrared spectroscopy
XRD	X-ray diffraction
ATR-FTIR	Attenuated total reflectance–Fourier transform infrared spectroscopy
CLSM	Confocal laser light scanning microscopy
YMP	Yerba mate powder
AV	Aloe vera gel
TPS	Thermoplastic cassava starch
UV	Ultraviolet
AI	Artificial intelligence
